# Supplementary material for: Comprehensive Annotation and Functional Exploration of MicroRNAs in Lettuce
Source: Front Plant Sci. 2021 Dec 24;12:781836. doi: 10.3389/fpls.2021.781836 (PMC8739914; doi:10.3389/fpls.2021.781836)
Supplement: Supplementary file 2 [file Data_Sheet_8.ZIP › GSM2230747.plot/Lsa-miRN518_Lsat_1_v5_gn_8_48940.1_187_TPlot.pdf]

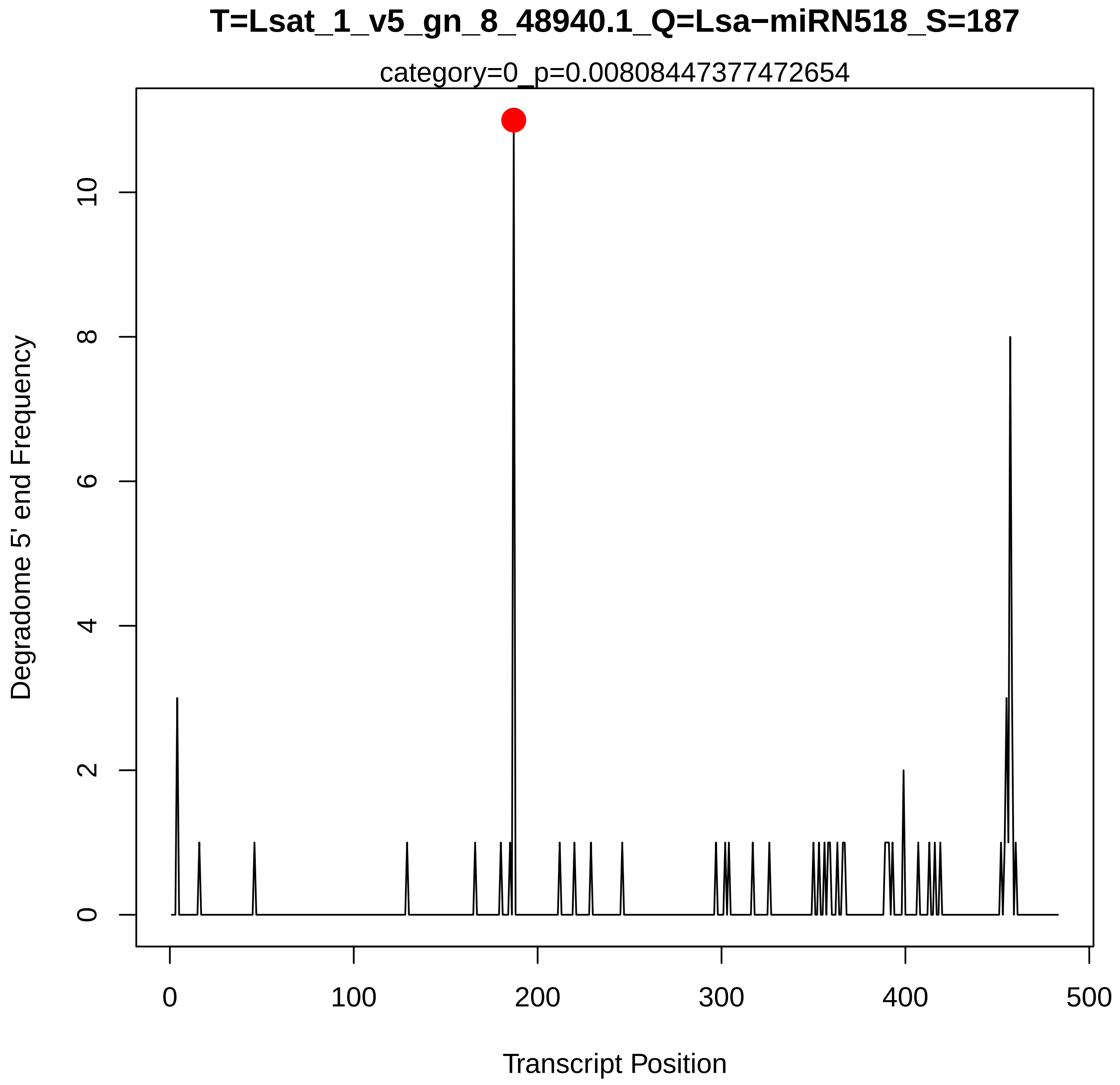

T=Lsat_1_v5_gn_8_48940.1_Q=Lsa−miRN518_S=187
category=0_p=0.00808447377472654
Degradome 5' end Frequency
0
2
4
6
8
10
0
100
200
300
400
500
Transcript Position
